# Mitochondrial aconitase and citrate metabolism in malignant and nonmalignant human prostate tissues

**DOI:** 10.1186/1476-4598-5-14

**Published:** 2006-04-04

**Authors:** Keshav K Singh, Mohamed M Desouki, Renty B Franklin, Leslie C Costello

**Affiliations:** 1 Department of Cancer Genetics, Roswell Park Cancer Institute, Buffalo, NY 14263, USA; 2 Department of Pathology Duke University Medical Center, Durham, NC 27710, USA; 3 Department of Biomedical Sciences, University of Maryland, Baltimore, MD 21201, USA

## Abstract

**Background:**

In prostate cancer, normal citrate-producing glandular secretory epithelial cells undergo a metabolic transformation to malignant citrate-oxidizing cells. m-Aconitase is the critical step involved in this altered citrate metabolism that is essential to prostate malignancy. The limiting m-aconitase activity in prostate epithelial cells could be the result of a decreased level of m-aconitase enzyme and/or the inhibition of existing m-aconitase. Earlier studies identified zinc as an inhibitor of m-aconitase activity in prostate cells; and that the depletion of zinc in malignant cells is an important factor in this metabolic transformation. However, a possibility remains that an altered expression and level of m-aconitase enzyme might also be involved in this metabolic transformation. To address this issue, the in situ level of m-aconitase enzyme was determined by immunohistochemical analysis of prostate cancer tissue sections and malignant prostate cell lines.

**Results:**

The immunocytochemical procedure successfully identified the presence of m-aconitase localized in the mitochondrial compartment in PC-3, LNCaP, and DU-145 malignant prostate cell lines. The examination of prostate tissue sections from prostate cancer subjects demonstrated that m-aconitase enzyme is present in the glandular epithelium of normal glands, hyperplastic glands, adenocrcinomatous glands, and prostatic intraepithelial neoplastic foci. Quantitative analysis of the relative level of m-aconitase in the glandular epithelium of citrate-producing adenomatous glands versus the citrate-oxidizing adenocarcinomatous glands revealed no significant difference in m-aconitase enzyme levels. This is in contrast to the down-regulation of ZIP1 zinc transporter in the malignant glands versus hyperplastic glands that exists in the same tissue samples.

**Conclusion:**

The results demonstrate the existence of m-aconitase enzyme in the citrate-producing glandular epithelial cells; so that deficient m-aconitase enzyme is not associated with the limiting m-aconitase activity that prevents citrate oxidation in these cells. The level of m-aconitase is maintained in the malignant cells; so that an altered enzyme level is not associated with the increased m-aconitase activity. Consequently, the elevated zinc level that inhibits m-aconitase enzyme is responsible for the impaired citrate oxidation in normal and hyperplastic prostate glandular epithelial cells. Moreover, the down-regulation of ZIP1 zinc transporter and corresponding depletion of zinc results in the increase in the activity of the existing m-aconitase activity in the malignant prostate cells. The studies now define the mechanism for the metabolic transformation that characterizes the essential transition of normal citrate-producing epithelial cells to malignant citrate-oxidizing cells.

## Background

In prostate cancer (PCa), malignancy develops mainly from the glandular epithelium of the prostate gland peripheral zone. A major and persistent characteristic that distinguishes normal prostate tissue from malignant prostate tissue is the extraordinarily high citrate content of the former versus the low citrate content of the latter [for reviews see [1-4]]. The normal secretory epithelial cells have the specialized function of production and secretion of extraordinarily high levels of citrate. To achieve this capability, these "citrate-producing" cells posses a unique limiting m-aconitase enzyme activity that impairs citrate oxidation. In malignant cells, m-aconitase activity is not limiting and citrate oxidation is not impaired. This metabolic transformation of normal citrate-producing cells to citrate-oxidizing malignant cells is an essential event in the development of prostate malignancy. Also, benign prostatic hyperplasia (BPH) involves the proliferation of citrate-producing glands. These are consistent relationships that have been corroborated and established by in situ magnetic resonance spectroscopy imaging of the human prostate; which is now the most reliable procedure for the identification and localization of malignant loci in the prostate gland [for reviews see [4-6]]. Consequently, it is essential to establish the mechanism of impaired citrate oxidation in the normal secretory epithelial cells, and the alteration of citrate production associated with the metabolic transformation in the malignant cells.

In normal mammalian cell intermediary metabolism m-aconitase typically exists in excess and is not a rate-limiting or regulatory enzyme, and catalyzes the equilibrium reaction:

**~88 citrate ←→ 4 cis-aconitate ←→ ~8 isocitrate**. 

This results in a characteristic citrate/isocitrate ratio ~10/1 for most mammalian tissues, regardless of the citrate concentration. In contrast, citrate-producing normal prostate glands and hyperplastic glands exhibit a citrate/isocitrate ratio ~30/1; which is indicative of a limiting m-aconitase activity [1,7]. This is substantiated by the impaired citrate oxidation but not isocitrate oxidation by citrate-accumulating prostate cells [8,9]. A limited m-aconitase activity could be the result of an inhibition of the enzyme and/or a decrease in the level of the enzyme. Previous studies established that the accumulation of high zinc levels, which occurs in normal prostate cells [3,10,11], results in the inhibition of m-aconitase activity and in a shift of its equilibrium toward citrate [12,13]. In PCa, the malignant prostate cells do not accumulate zinc, which leads to the expectation that m-aconitase activity is not inhibited in these cells and citrate oxidation occurs. In a recent study involving measurements with prostate cancer tissue sections (14), we demonstrated that ZIP1 zinc transporter is down-regulated and zinc levels are depleted in the glandular epithelium of adenocarcinomatous glands. However, an alternative or additional consideration is the possibility that the m-aconitase enzyme level might be low in citrate-producing normal prostate cells, and/or the enzyme might be over-expressed in malignant cells. Therefore, it was important to determine the level of m-aconitase enzyme in human prostate tissue samples and compare its level in malignant and nonmalignant glands. Moreover, this study was conducted with the same samples as in our previous report; so that the differences of changes in ZIP1 levels can be contrasted with the present results of m-aconitase levels. This report provides the first identification of m-aconitase enzyme in malignant and nonmalignant human prostate glandular epithelium.

## Results

**Figure 1:**
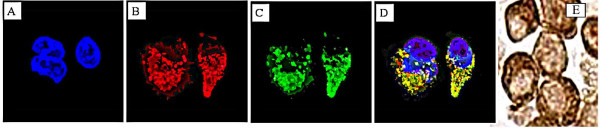
Subcellular localization of m-aconitase. **A-D**. Immunofluorescent detection. LNCaP prostate cell line was probed with Mitotracker then fixed for immunofluorescence with Aconitase-2 Ab and visualized by Laser Scanning Confocal Microscope. **A**. no staining of either in nuclei (DAPI). **B**. red punctuate mitochondrial staining in cytoplasm (Mitotracker). **C**. green fluorescent of m-aconitase in cytoplasm (FITC). **D**. red and green fluorescence merge shows co-localization of m-aconitase in mitochondria. **E**. Immunocytochemical detection of m-aconitase. Note the dark brown punctuate mitochondrial staining in cytoplasm; and well defined clear nucleus.

Costello et al [15] reported that the m-aconitase antibody employed in this present study was specific for the mitochondrial isoform and was not reactive with cytosolic (c-) aconitase. This conclusion was based on positive immunoreactivity (Western blots) with extracts of isolated mitochondrial preparations, and negative reactivity with cytosolic extract preparations. However, for the present immunohistochemistry studies, it was essential to establish that intra-mitochondrial m-aconitase could be detected and that the antibody reaction was limited to the mitochondria. To establish this, immunofluorescence analysis was conducted with LNCaP cells in conjunction with mitochondrial staining with Mitotracker. Figure 1 shows that the m-aconitase immunoreactivity was associated with and specific for the mitochondrial compartment, and that the antibody is selective for the m-aconitase isozyme.

**Figure 2:**
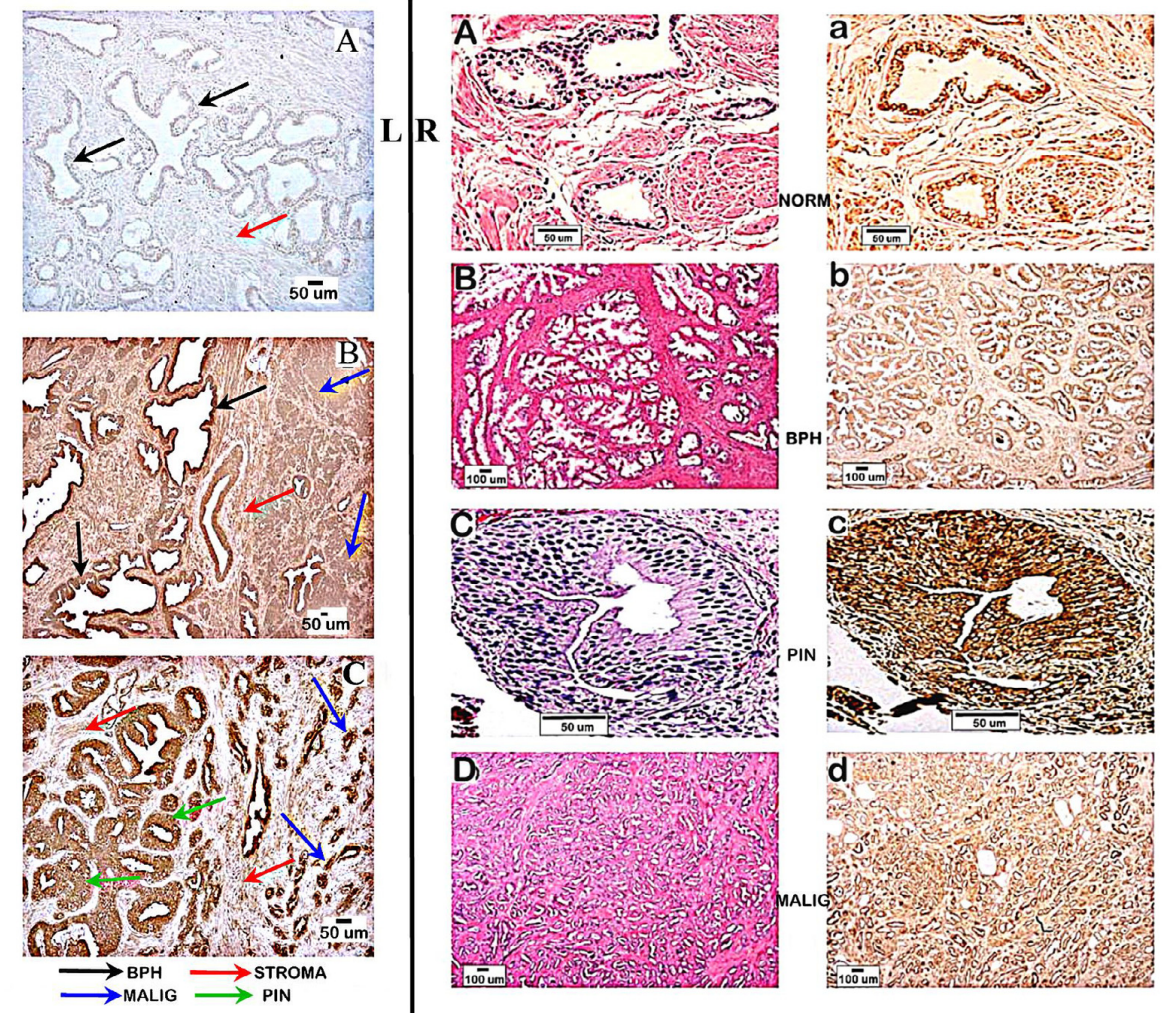
Representative m-aconitase immunohistochemistry of human prostate tissue sections. **Left panel**. **A**. Negative control. **B**. Field that shows adjacent BPH and malignant foci. **C**. Field showing adjacent PIN and malignant glands. **Right panel**. **A-D **are H&E staining; **a-d **are immunohistochemical staining. Note strong brown positive staining of glandular epithelium in all cases; and contrasting less staining of stroma.

We then proceeded to analyze human prostate tissue sections for m-aconitase immunoreactivity in malignant versus nonmalignant foci. Figure 2 presents the representative immunohistochemical detection of m-aconitase in BPH, malignant, normal, and PIN foci. The results show that m-aconitase is present in the glandular epithelium of all the glands regardless of the pathological state. Also, the cellular level of m-aconitase is consistently lower in stromal tissue than in glandular epithelium. Most importantly, immunopositive detection of m-aconitase is evident in the malignant glands as well as in the normal glandular epithelium.

Table 1 is the summary of the immunohistochemical scoring of m-aconitase reactivity of tissue sections from 22 cases of prostate cancer. The table presents the two parameters employed for quantitation of m-aconitase enzyme level: the percent of cells within a gland that exhibited m-aconitase immuno-positivity; and the cellular level of m-aconitase as represented by the m-aconitase-positive dots (mitochondria) within the cells. In this study, we restricted the analysis to the comparison of m-aconitase in glands located in the same tissue section. One reason was to eliminate, or at least minimize, any potential differences arising from the antibody diffusion into the cells and into the mitochondria for immunoreactivity. Thus, any comparative differences observed in the level of immunoreactivity in the different glands of the same tissue section would be due to comparative differences in the level of m-aconitase.

**Table 1:** Aconitase immunoreactivity of BPH glandular epithelumversus adenocarcinomatous glands of prostate cancer tissue sections.

	TUMOR	ACONITASE IMMUNOREACTIVITY		ZIP1 IMMUNOREACTIVITY	
CASE #	GRADE	BPH	PCA	BPH	Malignant

1	3	+++ 8.9	++ 6.4	++	Negative
2	3	+ 6.4	+++ 11.0	++	+
3	1	+ 6.2	+ 7.0	+	Negative
4	2	+++ 14.6	+++ 12.2	+	Negative
5	2	++ 12.6	+ 14.2	Negative	Negative
6	1	+++ 12.2	+++ 16.6	++	Negative
7	2	++ 10.0	+++ 15.6	+	+
8	1	+++ 11.4	++ 8.3	++	+
9	1	+ 7.5	+++ 18.0	++	Negative
10	2	+ 8.4	+ 8.6	++	Negative
11	1	+ 12.6	++ 11.8	++	Negative
12	2	+ 9.7	+++ 15.8	Negative	Negative
13	1	+++ 13.7	+++ 14.2	++	+
14	1	+++ 13.9	+++ 14.5	+	Negative
15	1	+ 7.6	+ 10.2	+	Negative
16	1	++ 11.6	+++ 13.9	+	Negative
17	1	++ 14.2	+++ 16.3	++	+
18	1	++ 9.2	++ 10.0	+	Negative
19	1	+++ 12.0	++ 10.9	+	Negative
20	2	+++ 14.7	+++ 18.2	++	+
21	1	+++ 14.1	+++ 17.6	Negative	Negative
22	3	+++ 16.3	+++ 17.9	-------------	-------------
t-TEST- MEAN(SE)					
POSITIVITY		2.1(0.85)	2.4(0.94)**	1.68(0.23)	0.32(0.10)*
QUANTITATION		11.3(2.9)	13. 1(3.8)**	-------------	-------------

Scoring of immunoreactivity: Negative, no positive cells;

score +, <10% positive cells; score ++, 10–50% positive cells;

score +++, > 50% positive cells.

Quantitation = Average number of well defined ACON IHC dots per cell.

Mean Positivity = sum of +'s/22

ZIP1 data taken from Franklin et al [14].

BPH vs Malignant *P < 0.01; **no significant difference

It is apparent from Table 1 that m-aconitase immunoreactivity is essentially the same for BPH glandular epithelium and adenocarcinomatous glands. BPH glands, like normal peripheral zone glands are citrate-producing glands; whereas adenocarcinomatous glands are citrate-oxidizing glands. Also, there is no correlation between the tumor grade and m-aconitase immunoreactivity. This is consistent with the fact that the decrease in citrate in malignant cells occurs very early in malignancy and persists through the progression of malignancy [4-6]. Although the number of cases in which tissue samples that contained normal glands and PIN (believed by many to be a precursor stage of malignancy) was insufficient for statistical analysis, these glands exhibited m-aconitase scoring similar to each other and similar to BPH and adenocarcinoma. These results show that alteration in the level of m-aconitase enzyme (i.e. altered m-aconitase expression/biosynthesis) is not associated with the altered citrate oxidation or production in malignant versus non-malignant cells. This is in contrast to our earlier study [14] of changes in the level of ZIP1 transporter in sections from the same tissue samples employed in this current report. For comparison, Table 1 shows those earlier results. The absence of a change in m-aconitase enzyme level is evident in the same glands that exhibit a marked down-regulation of the level of ZIP1 transporter in malignant versus non-malignant glands. Moreover, accompanying the down-regulation of ZIP1 is a corresponding decrease in cellular zinc levels [14]. Thus the absence of a demonstrable change in m-aconitase enzyme level is not due to some artifact effect.

**Figure 3:**
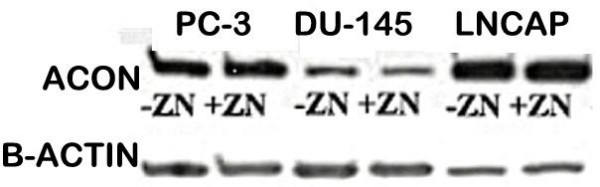
Western blot analysis of m-aconitase levels in human prostate cancer cell lines. The cells were exposed for 24 hours to medium supplemented with 15 uM zinc and to unsupplemented medium.

For additional corroboration of these results, we determined the expression of m-aconitase in LNCaP, DU-145, and PC-3 malignant prostate cell lines and the effect of zinc treatment on the level of m-aconitase in malignant prostate cell lines. The results (figure 3) show that m-aconitase is expressed in all the malignant cell lines. It is notable that the level of m-aconitase in untreated LNCaP cells is higher than PC-3 cells although citrate oxidation by LNCaP cells is negligible as compared to high citrate oxidation by PC-3 cells [16,17]. This is due to the higher accumulation of zinc in LNCaP cells that inhibits m-aconitase activity [16]. Exposure of the cells to zinc had no effect on the level of m-aconitase (figure 3). However, the conditions employed results in cellular accumulation of zinc (18, 19), which inhibits m-aconitase activity and citrate oxidation. Therefore, these studies establish, for the first time, that it is the accumulation of inhibitory levels of zinc on m-aconitase activity, and not limiting levels of m-aconitase enzyme, that prevents citrate-oxidation in prostate cells.

## Discussion

In typical mammalian cell metabolism, citrate is an essential intermediate for oxidation via the Krebs cycle and subsequent ATP production from coupled phosphorylation. Citrate must be converted to isocitrate by the action of m-aconitase as the entry step for its oxidation via the Krebs cycle. It is important in the intermediary energy metabolism of mammalian cells that m-aconitase is not a limiting reaction that would essentially truncate the Krebs cycle. Therefore, the constituitive m-aconitase activity is typically in excess in mammalian cells, which insures an adequate flux of citrate to isocitrate for oxidation as follows:



However, in normal prostate epithelial cells citrate is predominantly an end-product of intermediary metabolism, rather than an utilizable intermediate as in most other cells. It is well established that citrate-producing prostate cells and their mitochondria readily oxidize isocitrate, but not citrate [7-9,20]. These collective observations establish that the accumulation of citrate in citrate-producing prostate cells is predominantly due to a limiting m-aconitase activity (for additional supporting evidence see [1-4,21]. Therefore, the possibility existed that the constituitive level of m-aconitase enzyme in prostate cells might be uniquely low and a contributing factor in the limited m-aconitase activity. However, several studies with rat prostate cells, pig prostate cells and human prostate cell lines consistently demonstrated that the expression and level of m-aconitase is not lower in citrate-producing cells as compared to that found in typical citrate-oxidizing cells [15,22-24]. While this prostate m-aconitase relationship has been established in animal studies and in cell culture studies, the level of m-aconitase enzyme in human prostate tissue had never been determined. The present studies reveal that the constituitive level of m-aconitase is essentially the same in BPH glandular epithelium and normal peripheral zone glandular epithelium (both being citrate-producing glands), and the adenocarcinomatous glands (citrate-oxidizing glands) and PIN (presumed early malignant stage).

The absence of an altered expression of m-aconitase is in marked contrast with changes in the expression of ZIP1 zinc uptake transporter and in cellular zinc levels that we recently reported [14]. The elimination of the involvement of altered m-aconitase enzyme level leads to the conclusion that the rate-limiting m-aconitase activity is due to an inhibition of the enzyme. It is well established that normal peripheral zone glands and BPH glands accumulate extremely high zinc levels in association with their unique ability to produce and accumulate extremely high citrate levels; and that, in PCa, the malignant glands exhibit a marked decrease in zinc levels and in citrate levels [10,11]. These relationships are consistent with the established effects of zinc in the inhibition of m-aconitase activity and citrate oxidation [12,13], which permits the unique peripheral zone glandular function (as in the prostate of other animals) of net citrate production. In PCa, the lost ability of the malignant cells to accumulate zinc eliminates its inhibition of m-aconitase activity; and thereby increases the oxidation of citrate and decreases the cellular level of citrate.

## Conclusion

The present study demonstrates the existence of similar levels of m-aconitase enzyme in non-malignant citrate-producing normal and BPH glandular epithelial cells as compared to the citrate-oxidizing malignant cells. Consequently, the limiting m-aconitase activity of the citrate-producing epithelial cells is not the result of a deficient level of the enzyme (reaction 1); and the increased m-aconitase activity of citrate-oxidizing cells is not the result of an elevated level of the enzyme (reaction 2), as follows:





These results, in concert with previous reports, establish the mechanism of the regulation of m-aconitase and citrate oxidation in normal human prostate glandular epithelial cells and in the metabolic transformation in the malignant cells. The inhibitory effect of elevated zinc levels on m-aconitase activity is responsible for the impaired citrate oxidation by normal and BPH citrate-producing prostate cell (reaction 1). The lost ability of the malignant cells to accumulate zinc removes the inhibition of existing m-aconitase so that citrate oxidation occurs (reaction 2). This elucidation of the mechanism associated with the metabolic transformation to citrate oxidation provides the bioenergetic and synthetic requirements that are essential for the manifestation of the malignant process of the prostate neoplastic cell. Thus, important insight into the genetic/metabolic relationships of the pathogenesis and progression of prostate cancer is now evolving, which will provide new approaches to its detection and treatment.

## Methods

### Human prostate tissue

Twenty-two cases of prostatic adenocarcinoma slides were obtained from RPCI that contained both adenocarcinomatous foci and adjacent benign prostatic hyperplasia. Four of the cases contained normal prostatic glands and six cases contained prostatic intra-epithelial neoplastic foci (PIN). One section of normal and another BPH were obtained from the Cooperative Human Tissue Network of the National Cancer Institute, National Institutes of Health, Bethesda, MD. The slides were coded without identification related to the patients. Institutional Review Board approval was obtained.

### Immunohistochemistry

Immunohistochemistry was performed with the m-aconitase antibody (1:500 dilution) developed and described by Costello et al [15]. The immunohistochemistry protocol is described by Desouki and Rowan [25]. Briefly, the slides were de-parafinized by incubation in xylene and ascending grades of alcohol. Antigen retrieval was done by heating in 10 mM sodium citrate buffer (pH 6.0) @ 98°C, incubated in 1% hydrogen peroxide, blocked with 10% goat serum with avidin D (Vector Laboratories, Burlingame, CA), incubated with m-aconitase antibody in 10% goat serum with biotin at 4°C over night followed by incubation with secondary peroxidase labeled anti-rabbit IgG (H+L) antibody (Vector Laboratories, Burlingame, CA) in a concentration of 5 ug/ml. Color was developed by incubating slides with DAB kit (Vector Laboratories, Burlingame, CA) followed by counterstaining with Hematoxylin. One section from each prostatic tissue was stained with H&E for histological characterization of lesions and grading of tumors by a pathologist (M.M.D), according to the World Health Organization grading system [26]. Grade 1 is defined by well-differentiated glands with minimal anaplasia in which the nuclei are almost uniform with minimal variation in size and shape and few detectable nucleoli. Grade 2 is defined by moderately differentiated glands with moderate nuclear anaplasia with many nucleoli. Grade 3 is defined by poorly differentiated or undifferentiated glands showing marked anaplasia in which the nuclei showed marked variation in size and irregular shapes, vesicular, with marked abnormal mitotic figures. All sections were examined with light E600 Nikon microscope. The pictures were processed with Spot software, version 4.1, Diagnostic Instruments, Inc (Sterling Heights, MI).

### Immunofluorescence localization of m-aconitase

LNCaP cells (human prostate cancer cell line) were grown on cover-slips and maintained in RPMI media with glutamine (Gibco, Grand Island, NY) supplemented with 10% FBS + 100 U/ml penicillin and 100 ug/ml streptomycin + 20 ng/ml EGF for immunofluorescence. The sub-cellular localization of m-aconitase in relation to mitochondria was determined by the combination of immunofluorescence with m-aconitase and the MitoTracker Red CMXRos selective probe (Molecular Probes, Eugene, OR). LNCaP cells were grown on the laminin-coated glass cover slips in tissue culture plates until reaching about 75% confluence. The immunofluorescence protocol described by Makino et al [27] was employed with modification. The cells were incubated in media containing 100 nM MitoTracker for 30 minutes at 37°C. The medium was then replaced with fresh media without MitoTracker and incubated for 15 minutes at 37°C. Fixation of the cells was done in freshly prepared media containing 4% paraformaldehyde for 15 minutes at 37°C. Permeabilization was performed with 0.2 % Triton-x100 in PBS for 5 minutes at room temp and blocking with 1% BSA in PBS for 1 hour at room temperature. The cells were washed with PBS and incubated with m-aconitase antibody for 60 minutes at room temperature; and followed by incubation with secondary anti-rabbit IgG-FITC (Covance Research Products, Inc. Denver, PA) in 1% BSA for 60 minutes (1:1000 dilution). Mounting with Vectashield with DAPI mounting media (Vector Laboratories, Burlingame, CA) was done before sealing with glass slides. Microscopic examination with Leica SP2 Laser Scanning Confocal Microscope was carried out. Emissions were; Blue Diole Laser 405, Argon Laser 488 and Helum/Neon Laser 543 for DAPI (blue), FITC (green) and MitoTracker (red), respectively.

### Evaluation of m-aconitase immunoreactivity

The m-aconitase enzyme level was determined by the immunohistochemistry scoring method of Desouki and Rowan [25]. Two criteria were employed: the percent of epithelial cells within the glands that exhibit m-aconitase immunopositivity; and the number of immunopositive cytoplasmic dots (aconitase-detected mitochondria) within the epithelial cells. The cells with punctuate cytoplasmic dots were considered positive for m-aconitase. Twenty to fifty randomized high power fields (oil immersion, x100) in a section from each case were evaluated. The scores employed were; negative, no positive cells, score +, <10% positive; score ++, 10–50% positive; score +++, > 50% positive. For quantifying the m-aconitase within the epithelial cells, the average number of identifiable well-defined m-aconitase immunopositive dots per cell was determined by oil immersion high power field (x100) examination of 50 cells. The Excel program was used to calculate the correlation between tumor grades and aconitase immunopositivity scores. A t-test was also conducted for statistical comparison of m-aconitase levels in BPH glands versus adenocarcinomatous glands.

### Western blot analysis of m-aconitase

The level of m-aconitase in the malignant prostate cell lines was determined by Western blot analysis of cell extracts as previously described [15].

## Authors' contributions

KS directed the study at Roswell Park Cancer Institute. MD conducted histopathological and immunohistochemical analyses of prostate cancer slides; and the immunofluorescent study of malignant prostate LNCaP cells. KS and MD provided the data from theses analyses.

RBF and LCC conceived the study, developed and provided the m-aconitase antibody; conducted the malignant cell line studies at the University of Maryland. All authors contributed to the writing of the manuscript; and read and approved the final manuscript
